# Characterizing Transcriptional Networks in Male Rainbow Darter (*Etheostoma caeruleum*) that Regulate Testis Development over a Complete Reproductive Cycle

**DOI:** 10.1371/journal.pone.0164722

**Published:** 2016-11-18

**Authors:** Paulina A. Bahamonde, Mark E. McMaster, Mark R. Servos, Christopher J. Martyniuk, Kelly R. Munkittrick

**Affiliations:** 1 Canadian Rivers Institute and Department of Biology, University of New Brunswick, Saint John, New Brunswick, Canada; 2 Environment Canada, Canada Center Inland Waters, National Water Research Institute, Aquatic Contaminant Research Division, Burlington, Ontario, Canada; 3 University of Waterloo, Department of Biology, Waterloo, Ontario, Canada; Shanghai Ocean University, CHINA

## Abstract

Intersex is a condition that has been associated with exposure to sewage effluents in male rainbow darter (*Etheostoma caeruleum*). To better understand changes in the transcriptome that are associated with intersex, we characterized annual changes in the testis transcriptome in wild, unexposed fish. Rainbow darter males were collected from the Grand River (Ontario, Canada) in May (spawning), August (post-spawning), October (recrudescence), January (developing) and March (pre-spawning). Histology was used to determine the proportion of spermatogenic cell types that were present during each period of testicular maturation. Regression analysis determined that the proportion of spermatozoa versus spermatocytes in all stages of development (R^2^ ≥ 0.58) were inversely related; however this was not the case when males were in the post-spawning period. Gene networks that were specific to the transition from developing to pre-spawning stages included nitric oxide biosynthesis, response to wounding, sperm cell function, and stem cell maintenance. The pre-spawning to spawning transition included gene networks related to amino acid import, glycogenesis, Sertoli cell proliferation, sperm capacitation, and sperm motility. The spawning to post-spawning transition included unique gene networks associated with chromosome condensation, ribosome biogenesis and assembly, and mitotic spindle assembly. Lastly, the transition from post-spawning to recrudescence included gene networks associated with egg activation, epithelial to mesenchymal transition, membrane fluidity, and sperm cell adhesion. Noteworthy was that there were a significant number of gene networks related to immune system function that were differentially expressed throughout reproduction, suggesting that immune network signalling has a prominent role in the male testis. Transcripts in the testis of post-spawning individuals showed patterns of expression that were most different for the majority of transcripts investigated when compared to the other stages. Interestingly, many transcripts associated with female sex differentiation (i.e. *esr1*, *sox9*, *cdca8* and *survivin*) were significantly higher in the testis during the post-spawning season compared to other testis stages. At post-spawning, there were higher levels of estrogen and androgen receptors (*esr1*, *esr2*, *ar*) in the testis, while there was a decrease in the levels of sperm associated antigen 1 (*spag1*) and spermatogenesis associated 4 (*spata4*) mRNA. *Cyp17a* was more abundant in the testis of fish in the pre-spawning, spawning, and post-spawning seasons compared to those individuals that were recrudescent while aromatase (*cyp19a*) did not vary in expression over the year. This study identifies cell process related to testis development in a seasonally spawning species and improves our understanding regarding the molecular signaling events that underlie testicular growth. This is significant because, while there are a number of studies characterizing molecular pathways in the ovary, there are comparatively less describing transcriptomic patterns in the testis in wild fish.

## Introduction

The freshwaters of North America are populated by a rich variety of native fishes. Research has been restricted to a few common species, and relatively little attention is given to other groups, including the darters (Family Percidae). Often these lesser-studied species can function as valuable indicators of ecological integrity, and can provide important information about the health status of freshwater systems. Teleost fishes, including darters, have diverse reproductive strategies. As such, fish monitoring programs have grouped species with different reproductive strategies into three major categories: single spawners, multiple spawners with relatively few spawning events, and multiple spawners that show multiple spawning events [1]. Rainbow darter (RBD, *Etheostoma caeruleum*) is a multiple spawner, and the few spawning events tend to occur in late spring and early summer. RBD is currently used as a sentinel species to monitor sewage effluent impacts in river systems in southern Ontario, Canada [2] and is gaining popularity for environmental monitoring practices.

Spermatogenesis is the process by which a large number of spermatozoa cells are produced from a small group of spermatogonia stem cells [3]. The process is primarily under the influence of steroids hormones [4]. Plasma levels of androgens (testosterone, T; 11-ketotestosterone, 11-KT) increase gradually as spermatogenesis proceeds, followed by a decrease in production at spermiation. These events are coordinated by environmental factors that include temperature and photoperiod [5]. Similar to most fishes, the RBD reproductive cycle is highly controlled by temperature [6]. Due to ranges in temperature zones, many species begin gonadal recrudescence in the fall, grow their gonads throughout the winter months, and become mature in early spring [6]. Growth of the gonad occurs from the regressed stage, characterized as ~0.1% of the body weight (GSI = 0.1[7]), to a fully mature stage (GSI ≈ 1.5). The spawning season for RBDs in the Grand River in southern Ontario occurs between April and June with the post-spawning season occurring between July and August [8]. Hence, the process of testis development in RBD is relatively well described throughout the breeding season. Transcriptomic-based studies have provided valuable insight into the molecular events that underlie gonad development and maturation. While there have been several studies directed towards understanding gonad development in female fishes [9–12], there has been less information regarding the molecular changes that occur in the testis. As an example, in male rainbow trout, follicle stimulating hormone, which stimulates the testis to undergo sperm maturation, increases the expression of genes related to fatty acid mobilization and metabolism, Wnt signaling (*wisp1*), tight junction transcripts (*claudins*), and proteases (*cathepsin L*) among others [13]. Thus, some processes are described in terms of regulation, but large-scale studies describing the developmental changes that occur in the testis transcriptome are not widely available.

The main objective of this study was to determine the pathways related to RBD testis development over an annual cycle in RBD. Fish were collected from the Grand River, Ontario, Canada, in an area considered low impact in terms of anthropogenic pollution. A custom second generation RBD microarray was used to determine the major pathways involved in the male reproductive cycle in relation to the relative proportion of sperm types in the testis. In addition, a suite of genes were examined in males over a compete breeding season using real-time PCR to determine optimal sampling times for molecular endpoints in monitoring programs based on variability.

## Methods

### Fish sampling

Adult male RBD were collected from the Grand River, ON, Canada from a site outside the urban areas and away from anthropogenic inputs (43° 30’ 17” N, 80° 28’ 28” W) using a backpack electrofisher (Smith-Root Model 12-D). All fish captured by electrofishing were held in aerial buckets with water from the river. After species identification, fish that were juveniles or other species were released. For the RBD lethal sampling, fish were anesthetized in 0.1 g/L tricaine methanesulfonate (MS-222), and killed by cervical dislocation. All wild fish were collected in conjunction with the University of Waterloo under the approved animal care AUPP 10–17 by University of Waterloo Office of Research Ethics.

RBD were measured for weight (± 0.001 g), length (± 1 mm), gonad weight (± 0.001 g) and liver weight (± 0.001 g). Morphological endpoints were used to calculate condition factor [k = 100 * (body weight/length3)], gonadosomatic index [GSI = 100 * (gonad weight/body weight)], and liversomatic index [LSI = 100 * (liver weight/body weight)]. Gonads were divided in half with one lobe collected and processed for histology (placed in Davidson’s solution) and one lobe collected for molecular studies (flash frozen in liquid nitrogen). Five field collections were conducted from May 2011 to March 2012 with the goal being to collect males with distinct stages of testis development (i.e. May: spawning; August: post spawning; October: recrudescence; January: developing and March: pre -spawning). The Grand River Conservation Authority provided water quality data and this is provided in S1 Table.

### Histological Analysis

Testis were excised and examined to determine developmental stage. A lobe of the testis was fixed in Davidson’s solution for 7 d and placed in 70% ethanol. The tissues were imbedded with paraffin, and sectioned with a microtome (Leica RM 2155) at 5μm thickness. The tissue sections were placed on the microscope slides, and stained with hematoxylin and eosin. Approximately 15 males per site were randomly selected to examine differences between the reproductive stages. All sections from each male fish were examined in a random fashion. An image at 40x magnification was collected and a 13x17 point grid was overlaid before saving the image (221 points total). Each cell type (spermatogonia, spermatocytes, spermatids, spermatozoa, and others) at each grid intersection was scored, and five random images per fish were analyzed (total of up to 1,105 cells/fish; Northern Eclipse (v8.0) software package (Empix Imaging, 2006)).

### Extraction of mRNA and preparation of cDNA

The QIAGEN® RNeasy Mini Kit (Qiagen, Mississauga, ON, Canada) was used to isolate total RNA from the testis according to the protocol from post-spawning gonadal tissues because they were ≤ 1mg. For other fish, total RNA was isolated using TRIzol reagent (RNA Isolation Protocol, Life Technologies, Burlington, ON, Canada). Between 5 to 50 mg of tissue was homogenized with 1 ml of TRIzol reagent using the VWR® VDI 12 Adaptable Homogenizer as per our methods [14]. All RNA was purified through a QIAGEN® RNeasy Mini column prior to real-time PCR and microarray analysis, thus every sample was column purified. RNA concentration and A260/A280 ratio (between 1.8 and 2.0) was measured using the NanoDrop™ 2000 (Thermo Scientific, Canada). RNA integrity was evaluated using the 2100 Bioanalyzer (Agilent) with the RNA 6000 Nano kit. The integrity of total RNA, as measure by the RNA Integrity Number (RIN) was between 9.2 and 10 for all samples. RNA was stored in DEPC water at −80°C prior to microarray and gene expression analysis.

DNase treatment of 250 ng RNA was performed with TURBO DNA-free (Ambion, Burlington, ON, Canada) according to the manufacturer’s protocol for real-time PCR. Reverse transcription for cDNA synthesis was performed with SuperScript II Reverse Transcriptase (Life Technologies, Canada) using established protocols [15]. The cDNA samples were kept at -20 °C until used for real-time PCR.

### SYBR green Real-time PCR

All primers for real-time PCR were designed using Primer3 [16] (http://biotools.umassmed.edu/bioapps/primer3_www.cgi) and were synthesized by Life Technologies (Burlington, ON, CAN). Optimal annealing temperature for primers was between 58° and 60°C. Four control genes were selected for real-time PCR reactions: *rps18* (sequence found in Bahamonde et al. [15]), and *rps7*, *rps12*, and *rps40*. These reference genes were selected here because these targets did not change in relative abundance over the reproductive stages. Primer sequences were as follows: *Rps7* forward GCTCTCGGCAAATATGGC and reverse TCTCCTCCCTCCACAAAGTG (E = 108.1%, R^2^ = 0.98); *rps12* forward TTAGCTTGAAACCCGAAGGA and reverse ACACCTCGACCTGACGTTCT (E = 110.8%, R^2^ = 0.882); *rps40* forward TCTGCAATCATGGGAAAGTG and reverse ACGAAGGCGGTGATCTTCT (E = 95.7%, R^2^ = 0.99). In addition, *spag* was a new transcript measured here and its primer sequence was forward GCTCGCCGTCTATTTGTGA and reverse CAGGATGTGTAGTGTGAACTGATG (E = 94.3%, R^2^ = 0.99).

To confirm that a single PCR product was amplified, a PCR reaction was performed as previously outlined [15] and amplicons were subjected to electrophoresis on an agarose gel (5%) to visualize the unique band with SYBR safe DNA gel stain and a Low DNA Mass Ladder (Invitrogen). The two step real-time PCR using a CFX96 BioRad instrument began with enzyme activation at 95°C for 30 s, followed by 39 cycles of 5 s at 95°C and 30 s at the specific annealing temperature. The reaction was then measured at increasing temperatures in increments of 0.5°C from 65°C to 95°C, every 5 s to generate a dissociation curve. There were 7–9 biological replicates for each reproductive stage, and each sample was analyzed in duplicate. Each gene plate also contained a 6-point standard curve in duplicate. In addition, two no template control (NTCs) and two no reverse transcriptase controls (NRT) were included as negative controls. The target stability value for the 4 control genes was M-value = 1.12 (CV = 0.47). Normalized gene expression was extracted using CFX Manager™ software and gene expression differences determined using the relative ΔΔCq method. All amplicons were verified as correct target genes by Sanger Sequencing at the McGill University and Génome Québec Innovation Centre (Montréal, QB, Canada). MIQE guidelines were carefully considered for real-time PCR analysis [17].

### Microarray development

We designed a new RBD microarray platform by selecting annotated genes from a first generation microarray [14], and this became a 8x15K second-generation microarray. Briefly, a total of 6,202 annotated probes were included in duplicate on the 8x15k Agilent platform. Additional positions on the array contained unknown probes that were added randomly to the second-generation array based upon the criterion that the probe showed relatively high expression in the testis (intensity > 7.5). The arrays were printed by Agilent Technologies (Santa Clara, CA, USA). The RBD microarray platform has been deposited into Gene Expression Omnibus (GPL18038, GSE57865).

### Gene expression analysis using the RBD oligonucleotide 8x15K microarray platforms

Gene expression analysis was performed on fish at five stages: recrudescence (n = 5), developing (n = 6), pre-spawning (n = 7), spawning (n = 7), and post-spawning (n = 6) (n = 31 microarrays). Microarray hybridizations were performed according to the Agilent One-Color Microarray-Based Gene Expression Analysis protocol using Cyanine 3 (Cy3) and 100 ng total RNA per sample was used for the production of cDNA and labeled/amplified cRNA as per the Agilent Low RNA Input Fluorescent Amplification Kit. Labeling methods followed those previously described [18]. Each RBD testis sample showed a specific activity >6.0 pmol Cy3/mL and amounts were adjusted to a final mass of 0.825 μg for 8x15K microarray hybridizations. Fragmentation of the cRNA, hybridization, and slide washes after the 17 h hybridization followed instructions outlined by Agilent (Gene Expression Hybridization Kit). An ozone barrier slide was used to cover microarrays before scanning. Microarrays were scanned at 5 μm with the high density Agilent DNA Microarray Scanner (Agilent Technologies). Agilent Feature Extraction Software (v9.5) scanned arrays with a full dynamic range. The quality of microarray data was evaluated by manual inspection and the quality control report generated by Feature Extraction Software (Agilent Technologies). Each microarray was deemed to be of high quality based on visual inspection.

### Bioinformatical analysis

Gene expression data were normalized using Quantile normalization [19]. Data were filtered prior to identification of differentially expressed genes (DEGs). All gene probes below an intensity of 3.5 were assigned a value of 3.5 as this was estimated to be the limit of detection of the microarray analysis (based upon the lower limit of the standard curve for Agilent spike in controls and negative controls across all microarrays). Approximately 5.5% of all spots across all microarrays fell below this intensity. This was a conservative limit for subsequent analyses to identify DEGs. Normalized data were subjected to hierarchical clustering in JMP Genomics to visualize global patterns of gene responses. Hierarchical clustering and k-means cluster analysis involved complete linkage and used normalized expression data. Functional enrichment of gene ontology terms and Principle Component Analysis (PCA) were performed in JMP Genomics (V6.0).

Pathway Studio 9.1 (Ariadne, Rockville, MD, USA) and ResNet 9.0 (Mammals) (Nikitin et al., 2003) were used for gene set enrichment analysis (GSEA) and sub-network enrichment analysis (SNEA). These two approaches were employed to gain additional insight into patterns of gene expression in testis development. GSEA is a method widely used in microarray analysis to determine if molecular pathways are enriched for specific annotated cell pathways [20]. SNEA analysis differs from GSEA in that sub-networks are built based upon the relationship of the regulated gene with other genes or proteins in the same biological pathway. Entities are ‘‘connected” to each other based upon relationships that are user-defined (i.e. binding partners, expression targets, protein modification targets, cell process). An algorithm compares the sub-network distribution of the regulated genes to the background distribution of other genes in the characterized interaction network. Thus, data mining of the ResNet 9.0 database yields interactions among molecular and cellular processes.

The GO term analysis was conducted using GOrilla (Gene Ontology enRIchment anaLysis and visuaLizAtion tool). We searched for enriched GO terms in the cluster list of genes compared to the background list of the total genes on the microarray. The algorithm recognized 5,838 genes out of the 11,762 gene terms that were entered. There were 4,316 duplicate genes that were removed (keeping the highest ranking instance of each gene), leaving a total of 1,522 genes.

### Statistical Analysis

Morphometric data were tested for equal variance (Levene’s test) and normality (Kolmogorov-Smirnov test). Log transformed data met the assumptions to test differences in fish length, body weights, condition factor (k), gonadosomatic index (GSI), and liversomatic index (LSI) using analysis of variance (ANOVA). A Tukey’s post hoc test identified individual stage differences. For the histological analysis, the 5 replicate micrographs per individual were pooled together, the data were arcsine transformed, and an ANOVA was used to test for differences among the individual responses across reproductive stages.

A Kruskal-Wallis test was performed on the Cq values of the raw expression data to eliminate any unsuitable reference genes that varied with reproductive stage. Following this, normalized gene expression data were extracted using CFX Manager™ software using a relative ΔΔCq method. The data were tested for equal variances and for normality. Differences among group means were tested by ANOVA (p < 0.05) on the log transformed expression data, followed by Tukey’s post hoc tests to identify if there were any differences between stages.

The k-means algorithm was used for the computation of different gene expression trends in the set of 9, 344 unique genes on the array in male gonads across the annual cycle. K-means clustering is an iterative procedure aimed to reduce the variance to a minimum within each cluster. An automated radius K-means was used with a correlation radius for clustering of 0.8. This yielded a total of 183 different patterns of gene expression over the different stages of development, and these were further pooled into 18 patterns of gene expression.

## Results

### Rainbow darter morphological characterization

**Fig 1:**
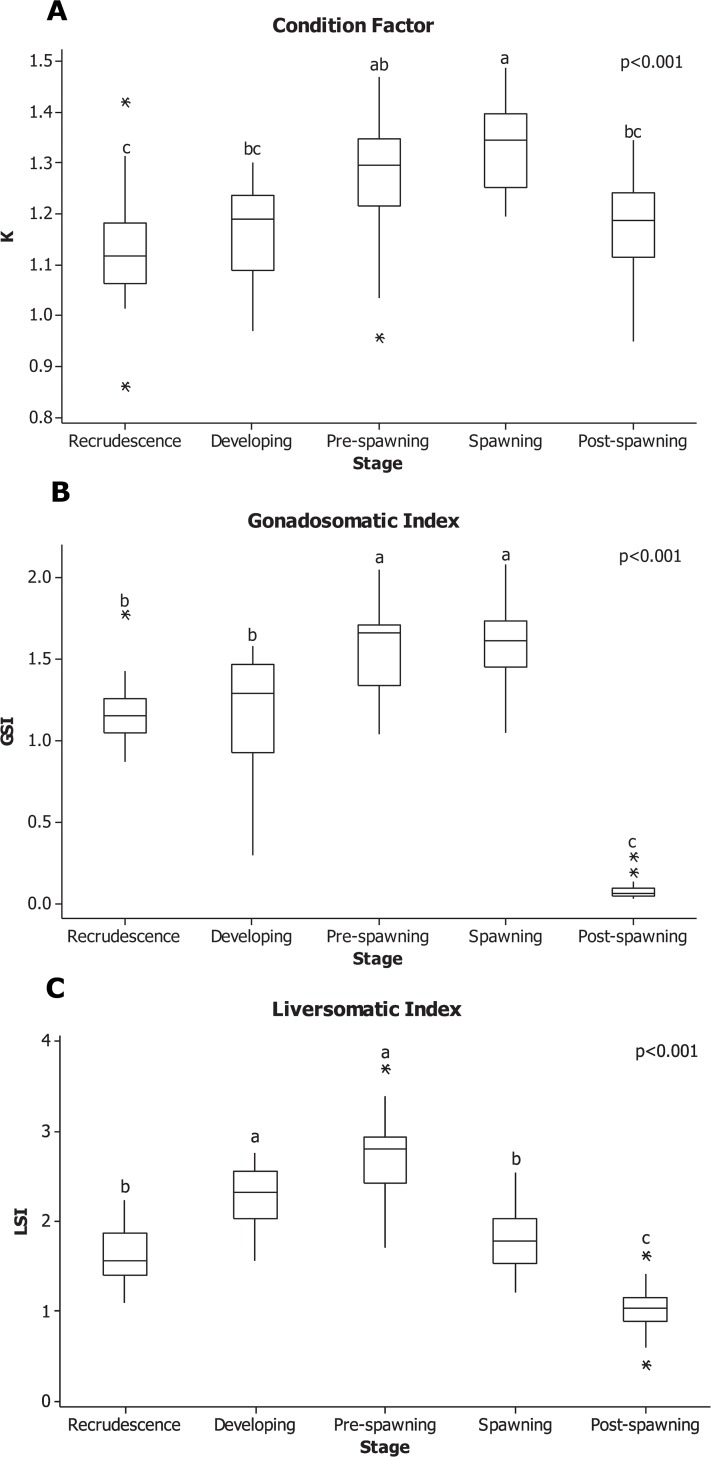
Boxplot of A. condition factor, B. gonadosomatic and C. liversomatic indices of male RBD over the different stages in an annual cycle. Fish were collected from May, 2011 to March, 2012. The horizontal line in box plots is the median, the boundaries of the box represent the 25th and 75th percentiles (boundaries of the box). The minimum and maximum data points are represented by the whiskers, and the outliers are represented by asterisks. Significant differences were test by ANOVA between stages and are indicated by different letters (p < 0.05).

All males that were collected were adults, with a minimum length of 4.00 cm and a maximum length of 7.20 cm. Fish from May (6.11 ± 0.09 cm) were larger than males collected in August (5.56 ± 0.10 cm) and October (5.38 ± 0.12 cm) (p = 0.02 and p < 0.001, respectively). In general, the males also collected in May were heavier (3.12 ± 0.17 g) than those collected in other seasons (p < 0.001), with individuals collected in the pre-spawning being an exception (2.45 ± 0.17 g; p = 0.10). This same pattern was also observed with condition factor (k). Males collected in May had the highest k (k = 1.33 ± 0.02) compared to all other seasons. The k of spawning males was different from males collected in the post-spawning (k = 1.18 ± 0.02; p < 0.001), recrudescence (k = 1.13 ± 0.02; p < 0.001) and developing (k = 1.17 ± 0.02; p < 0.001) period. No differences were detected between pre-spawning and spawning males (p = 0.20; Fig 1A).

As expected, the lowest mean GSI occurred when individuals were collected in the post-spawning stages (0.08 ± 0.01; p < 0.001) (Fig 1B). Shortly after post-spawning, the testis began to increase in size, reaching a peak GSI = 1.17 ± 0.05 in the pre-spawning and spawning period. The testis do not growing dramatically over the winter, and GSI was not significantly different in individuals collected between recrudescence and the developing stage (1.16 ± 0.09; p = 0.98). GSI increased in individuals at the pre-spawning season to 1.56 ± 0.06, and showed no difference when compared to males collected at the spawning season (1.59 ± 0.05; p = 0.99).

The LSI had a different pattern than GSI over the reproductive cycle (Fig 1C). Developing and pre-spawning males had the largest LSI (2.26 ± 0.09 and 2.75 ±0.09, respectively) compared to other males collected throughout the year. Males collected in the recrudescence and spawning season followed this had smaller LSI than developing and pre-spawning; finally, the males with the smallest LSI (1.02 ± 0.05) belonged to those at the post-spawning season compared to any other stage (p < 0.001).

### Histological analysis

**Fig 2:**
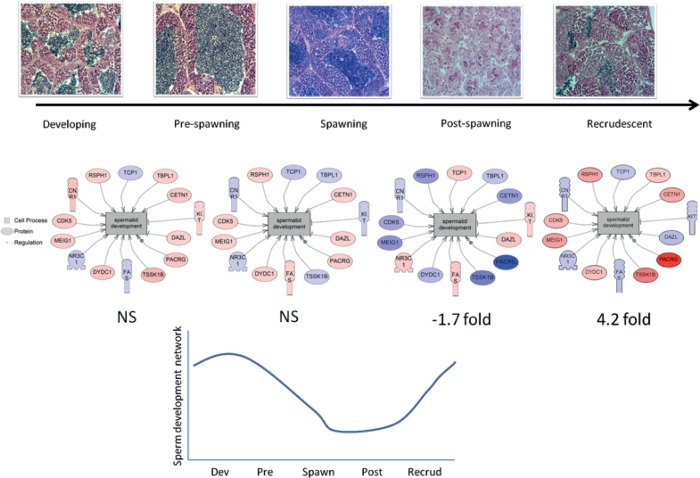
Testis structure from male rainbow darter during the periods designated as “developing, pre-spawning, spawning, post-spawning and recrudescence”. A. Cells are labeled as SG (spermatogonia), SC (spermatocytes), or ST (spermatozoa); the arrow represents 20 μm. B. Sub-network enrichment analysis of a spermatid expression network over testis development and a graph below representing the expression network over time based on fold changes of reproductive stage prior to the next acts as the relative comparison (i.e. from developing to pre-spawn, from pre-spawn to spawn, from spawn to post-spawn and from post-spawn to recrudescent). Blue colors represent a down-regulated gene and red colored figures represent an up-regulated gene. The curve represent the overall relatively abundance of the genes involved on the sperm development network. NS means no significant change

**Fig 3:**
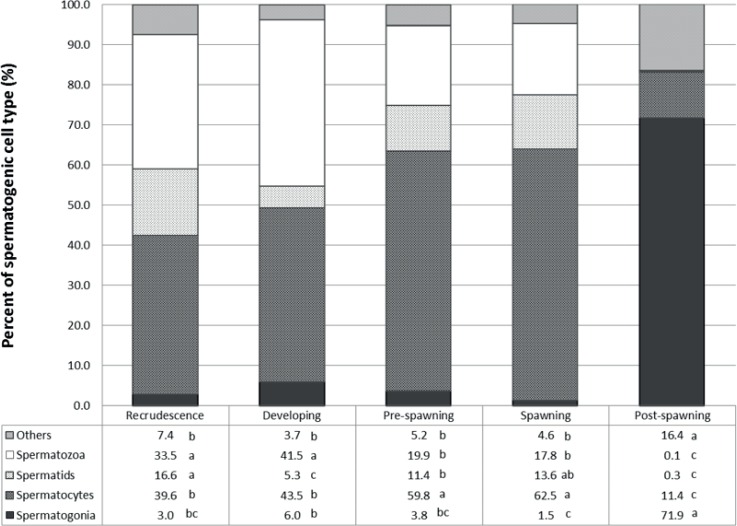
Proportions of gamete stages in the testes of male rainbow darter collected over an annual cycle. Letters signify a statistical difference between seasons within stages (p < 0.05).

Representative micrographs of the different stages of RBD testis development are shown in Fig 2A as well as a sperm maturation network (Fig 2B, described further below), and the spermatogenic cell type proportion in Fig 3. In the recrudescence stage, the major spermatogenic cell type present in the testis was spermatocytes (~40%). The cell type proportion between recrudescence and developing stage was not different except for a decrease in the proportion of spermatids (p < 0.001). However, the spermatogenic cell proportions for the next stage, pre-spawning, was very different compared to the others and the most dominant cell type proportion was spermatocytes (~60%), followed by spermatozoa (~20%). There was no statistical difference in gamete proportions in males between the pre-spawning season and the spawning season. The morphology and gamete proportions were also different in individuals collected in the post-spawning season. The major spermatogenic cell type at this stage of sexual development was spermatogonia (~72%), while the presence of spermatids or spermatogonia were almost completely absent. There was also a significant increase of non-gamete characteristics, primarily due to connective tissue and epithelial cells (~16%, p <0.001).

**Fig 4:**
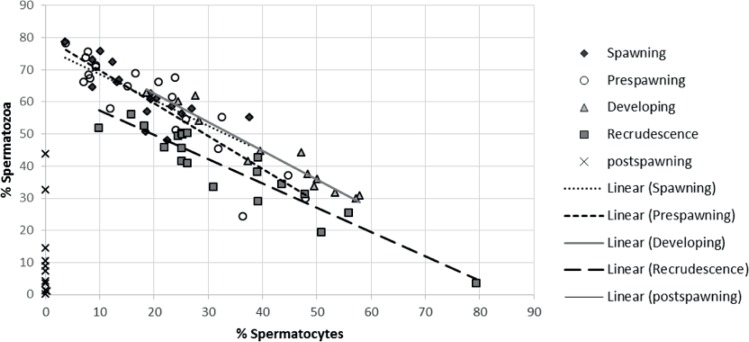
Linear regression between the percent of spermatozoa and the percent of spermatocytes. The equations of the trend lines are for four of the different stages of the annual cycle; recrudescence: y = -0.7566x + 64.882, R^2^ = 0.8791; developing: y = -0.895x + 80.694, R^2^ = 0.934 pre-spawning: y = -1.0205x + 80.027; R^2^ = 0.7977; and spawning: y = -0.803x + 76.75, R^2^ = 0.5874. Post-spawning fish were taken out of this analysis because they do not have the spermatozoa cell type.

The strongest relationship detected was between the % spermatocytes and % spermatozoa, which were correlated throughout the reproductive process (Fig 4). Cell types in post-spawning males did not correlate to other gamete stages due to the absence of spermatozoa. The increased proportion of spermatocytes was correlated with the decrease of spermatozoa at recrudescence (r^2^ = 0.88), developing (r^2^ = 0.93), pre-spawning (r^2^ = 0.80) and spawning (r^2^ = 0.59) seasons. No relationship was found between GSI and the proportion of any of the spermatogenic cell types (data not shown).

### Hierarchical cluster analysis and Principal component analysis (PCA)

**Fig 5:**
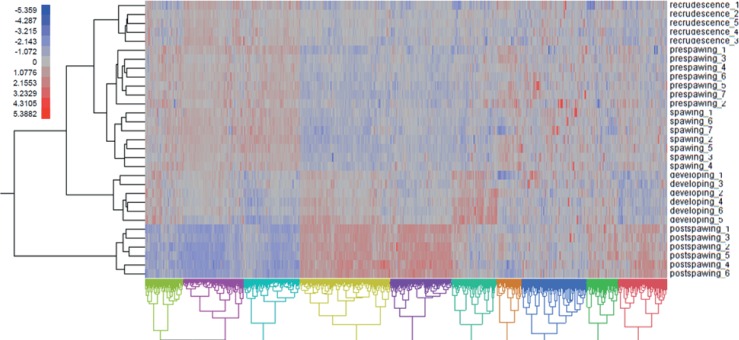
Hierarchical clustering of differentially expressed genes (p < 0.05) in the male gonad of rainbow darter over the different stages of development (i.e. recrudescence, developing, pre-spawning, spawning and post-spawning). Each row numbered represents different individual fish within a treatment; each column represents a specific gene. Co-expressed genes were classified in 10 major clusters (from A to J). Blue indicates low mRNA levels (relative) and red indicates high mRNA levels (relative).

Hierarchical clustering of the differentially regulated genes showed that expression patterns were strongly associated to the specific stages of gonad development (Fig 5). Expression profiles from males in pre-spawning and spawning appeared to be most closely related compared to other transcriptomics profiles. Post-spawning fish also showed a transcriptomics profile that was most different from the other stages, and this stage also showed the most differences among stages in terms of cell proportion composition. The analysis of the co-expressed genes (columns) showed 10 different expression clusters (from A to J, Fig 5).

**Fig 6:**
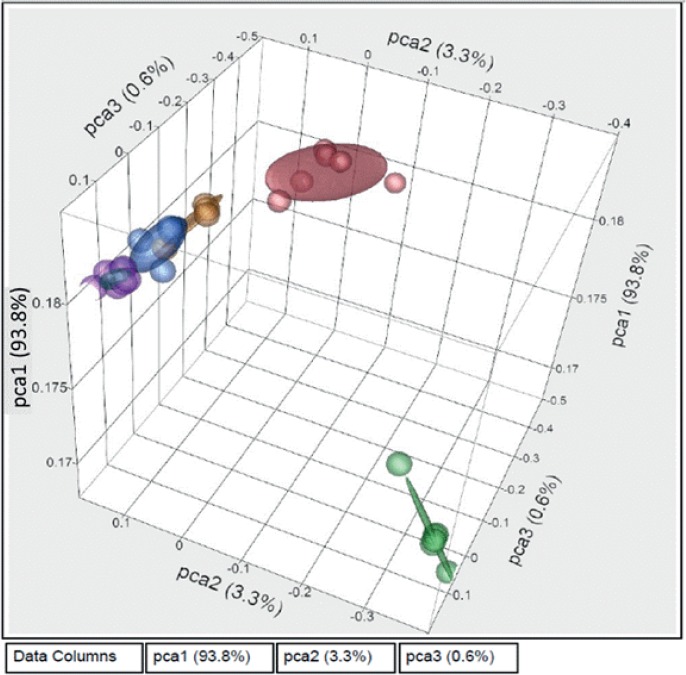
A 3D Principal Component Analysis of male rainbow darter gene expression profiles in gonad tissue across the annual cycle. Different colors represent the different stages of gonad maturity, including post-spawning (green at bottom right), recrudescence (yellow to the right of the cluster at top left), developing (red top right), pre-spawning (blue in the centre of the cluster at top left) and spawning (purple top left).

The first component of PCA analysis explained 93.8% of the variability in the expression data. PCA analysis showed the same pattern as that of the cluster analysis; males that were spawning, pre-spawning, and recrudescent showed a gene expression pattern that was most similar (Fig 6), while the developing males had a different distribution pattern, and the post-spawning males were most different from all other male stages.

### K-means cluster analysis

**Fig 7:**
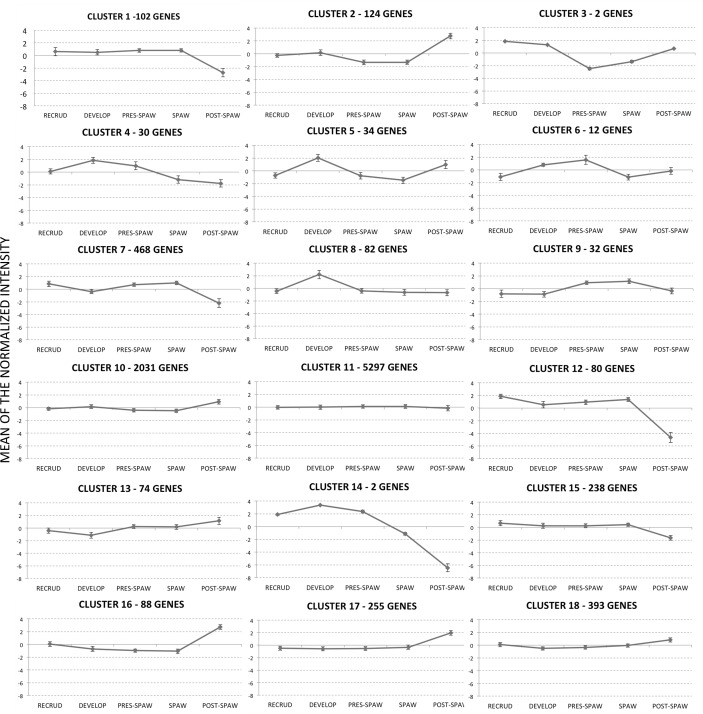
Gene expression patterns based upon k-means clustering. Data were used for the computation of expression trends in the set of 9,344 genes in male gonads across the annual cycle. K-means is an iterative procedure aimed to reduce the variance to a minimum within each cluster. A total of 18 different patterns of gene expression were determined. The tendency curve (centroid) is depicted by the solid line with the SE of the normalize intensity.

To identify the major temporal patterns of genes expression over the five stages of development, we performed a *K*-mean cluster analysis. An automated radius K-mean with a correlation radius for clustering of 0.8 resulted in a total of 183 patterns of gene expression over the different stages of development. Due to the large number of clusters, a manual inspection was applied to collapse the overall number of clusters. The data were reanalyzed by automated K-means with a total of 18 clusters that had similar patterns of gene expression or “waves” (S2 Table, Fig 7). The cluster with the highest number of genes was cluster 11 (5,297 transcripts), a cluster that contained a high proportion of genes that showed “no change” in relative expression over testicular development. The clusters that contained genes with the highest expression changes over testis development were 3 and 14. However, the large majority of genes that belonged to those clusters were not annotated, and we were unable to determine the biological processes that underlie those clusters.

### GO term and SNEA/GSEA analysis within expression clusters

There were two approaches used to identify unique gene networks within each testis stage transition. We first took a targeted approach to determine what major functions were enriched within the genes lists that belonged to each one of the 18 clusters identified by k-means clustering (Fig 7). The rationale was that these expression waves included genes involved in specific processes. However, we were not able to perform this analysis for all clusters due to the fact that some clusters had too few genes annotated. Table 1 list the GO terms enriched at each cluster analyzed. Cluster 4, for example, was characterized by transcripts that were increasing in abundance between the developing and pre-spawning season, followed by a decrease in abundance of the transcripts at the spawning and post-spawning season (Fig 7). This cluster had enriched GO terms that were related to lipid pathways. Cluster 10 and 11, which contained genes that showed no change in abundance over testis development, included enriched pathways associated with ribosomal proteins and cell cycle (Tables 1 and 2). Cluster 12 contained transcripts with high expression at the recrudescence, pre-spawning and spawning stages, followed by a decrease at post-spawning. This cluster contained transcripts that had functions related to reproduction.

**Table 1:** The most significant Gene Ontology (GO) terms enriched in K-means clustering.

Cluster	GO term	Description	P-value	FDR q-value	Enrichment (N, B, n, b)
4	GO:0034364	high-density lipoprotein particle	1.99E-04	1.55E-01	82.69 (1447,5,7,2)
	GO:0034358	plasma lipoprotein particle	2.98E-04	1.16E-01	68.90 (1447,6,7,2)
	GO:0032994	protein-lipid complex	2.98E-04	7.73E-02	68.90 (1447,6,7,2)
7	GO:0007268	synaptic transmission	3.60E-04	1.00E+00	6.71 (684,10,51,5)
10	GO:0003723	RNA binding	5.72E-05	8.23E-02	1.52 (1447,138,413,60)
	GO:0008135	translation factor activity, nucleic acid binding	2.50E-04	1.80E-01	2.28 (1447,23,413,15)
11	GO:0005654	nucleoplasm	5.24E-05	0.0407	1.20 (1447,170,960,135)
	GO:0044391	ribosomal subunit	0.000467	0.181	1.29 (1447,62,960,53)
	GO:0044445	cytosolic part	0.000482	0.125	1.28 (1447,66,960,56)
	GO:0015934	large ribosomal subunit	0.000738	0.143	1.37 (1447,34,960,31)
	GO:0044428	nuclear part	0.000903	0.140	1.11 (1447,345,960,253)
12	GO:0048610	cellular process involved in reproduction	6.05E-04	1.00E+00	9.41 (1447,41,15,4)
15	GO:0033365	protein localization to organelle	9.67E-04	1.00E+00	8.18 (1447,12,59,4)
18	GO:0051117	ATPase binding	3.51E-04	5.05E-01	14.05 (1447,3,103,3)

Not all clusters contained sufficient numbers of genes for analysis. 'P-value' is the enrichment p-value. This p-value is not corrected for multiple testing of 1438 GO terms. Enrichment (N, B, n, b) is defined as follows: N—is the total number of genes; B—is the total number of genes associated with a specific GO term; n—is the number of genes in the top of the user's input list or in the target set when appropriate; b—is the number of genes in the intersection.

**Table 2:** Sub-Network enrichment analysis (SNEA) of the proteins/chemical cell processes involved in each cluster.

Cluster	Proteins/Chemicals Regulating Cell Processes	Overlap genes	p-value
**2**	cell differentiation	8	<0.001
**2**	apoptosis	6	0.039
**2**	cell death	5	0.019
**7**	cytokinesis	13	0.003
**7**	spermatogenesis	8	0.008
**7**	meiosis	8	0.008
**7**	spermatid development	6	<0.001
**7**	membrane fusion	6	0.002
**7**	spindle assembly	6	0.043
**10**	apoptosis	156	<0.001
**10**	cell growth	106	<0.001
**10**	cell death	100	<0.001
**10**	cell cycle	92	<0.001
**10**	cell survival	68	<0.001
**10**	oxidative stress	59	<0.001
**10**	translation	53	<0.001
**10**	DNA Damage	43	<0.001
**10**	RNA splicing	42	<0.001
**10**	mitochondrial damage	38	<0.001
**10**	S phase	37	<0.001
**11**	apoptosis	352	<0.001
**11**	cell proliferation	317	<0.001
**11**	cell growth	257	<0.001
**11**	cell cycle	218	<0.001
**11**	cell death	215	<0.001
**11**	cell survival	147	<0.001
**11**	DNA replication	130	<0.001
**11**	mitosis	118	<0.001
**11**	oxidative stress	97	<0.001
**11**	RNA splicing	97	<0.001
**11**	translation	97	<0.001
**11**	DNA Damage	95	<0.001
**11**	DNA repair	80	<0.001
**13**	ROS generation	6	0.003
**13**	virulence	5	<0.001
**15**	cytokinesis	9	0.003
**15**	meiosis	6	0.005
**15**	virulence	5	0.01
**15**	immunoreactivity	5	0.012
**16**	cell death	7	0.026
**16**	cell growth	7	0.044
**17**	oxidative stress	12	<0.001
**17**	immune response	10	0.02
**17**	vascularization	10	0.035
**17**	contraction	9	0.011
**17**	ROS generation	8	0.024
**17**	translation	8	0.031
**17**	cytoskeleton organization and biogenesis	7	0.015
**17**	epithelial to mesenchymal transition	5	0.033
**17**	locomotion	5	0.034
**18**	ROS generation	14	0.004
**18**	oxidative stress	13	0.015
**18**	translation	13	0.017
**18**	calcium ion homeostasis	11	0.036
**18**	blood clotting	10	0.003
**18**	exocytosis	10	0.024
**18**	Respiratory chain	9	0.013
**18**	life span	9	0.017
**18**	vasodilation	7	0.026
**18**	mitochondrial membrane permeability	6	0.002
**18**	lipid peroxidation	6	0.004
**18**	endothelial cell function	6	0.027
**18**	Steroid metabolism	6	0.03
**18**	oxidative phosphorylation	5	0.002
**18**	fatty acid metabolism	5	0.004
**18**	sperm motility	5	0.007
**18**	response to hypoxia	5	0.01
**18**	gluconeogenesis	5	0.028
**18**	reproduction	5	0.031

Sub-network enrichment analyses (SNEA) were conducted for 9 different k-mean clusters (Table 2) to determine if there were specific biological entities enriched in each group of genes included within a cluster. For example, cluster 2 (Fig 7), which showed the lowest expression for transcripts at pre-spawning and spawning, followed by a peak at post-spawning, contained genes that were related to the cell process of apoptosis, cell differentiation, and cell growth (Table 2). Using the same analysis, clusters 10 and 11 contained genes that showed no change in abundance over the stages of development, and these transcripts tended to be involved in cell cycle and cell functions within the nucleus (Table 2). Genes in clusters 7 and 15 showed similar expression patterns of low abundance at post-spawning (Fig 7) and both clusters had an enrichment of cell processes related to spermatogenesis and meiosis (Table 2). Finally, genes within clusters 13, 17 and 18 shared a common pattern (Fig 7), with low expression at developing stages and a peak at post-spawning (Fig 7). SNEAs of these clusters showed and enrichment of genes related to immune system functions, in addition to lipid, fatty acid, and steroid metabolism. Cluster 18 also contained a subset of genes involved in sperm mobility.

**Fig 8:**
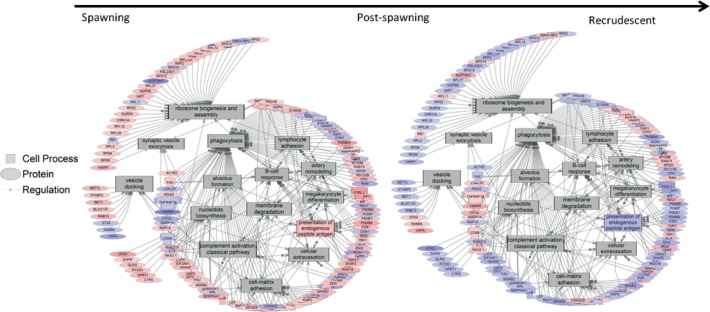
Processes activated in the transition from spawning to post-spawning. These processes are then suppressed as the testis enters recrudescence and begins a new maturation cycle. Blue colors genes represent a down-regulated gene and red colored figures represent an up-regulated gene.

Spermatid developmental pathways were investigated using pathway analysis to determine how genes related to this process changed from stage to stage (Fig 2B). The analysis indicated that there were no significant differences in the overall expression of genes that belonged to the spermatic development pathway from developing to spawning stages. However, there was a significant decrease in the expression of genes in this pathway from the spawning season to the post-spawning season (-1.7 fold change; p = 0.003). In addition, there was a significant increase in this pathway from the post-spawning season to the recrudescence stage (4.2 fold change; p ≤ 0.001). GSEA revealed several cell processes that changed over the reproductive cycle. Fig 8 shows that processes related with phagocytosis increased after the spawning season towards post-spawning season. In addition, the data suggest that cell processes involved in the immune system increased in fish at the post-spawning stage of development, which is consistent with the SNEA results (Table 2) of cluster 17 (Fig 7, peak abundance at post-spawning season).

**Fig 9:**
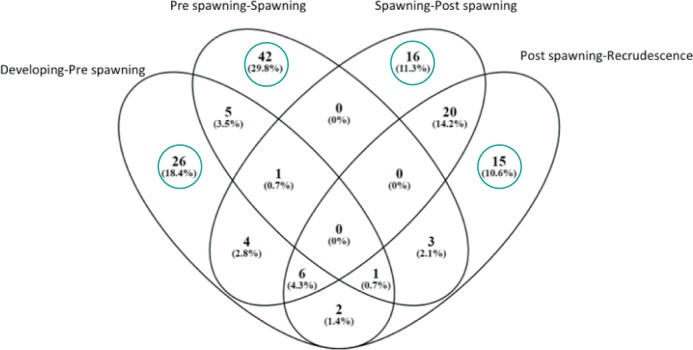
Venn diagram comparing sub-networks that were significantly expressed (p < 0.05) at each stage of testes development. Circled are the processes that are unique to each stage. These are presented in Table 3.

A second approach was employed to identify unique gene networks specific to the stage transitions. Venn diagram showed the number of genes that are significantly expressed (p < 0.05) at each stage of testes development (Fig 9) and they are later listed at Table 3. Enriched cell process that were affected when transitioning from Developing to Pre-spawning stages included nitric oxide biosynthesis, response to wounding, sperm cell function, and stem cell maintenance. The Pre-spawning to Spawning transition was characterized by gene networks related to amino acid import glycogenesis, Sertoli cell proliferation, sperm capacitation, and sperm motility. The Spawning to Post Spawning transition contained unique gene networks associated with chromosome condensation, endothelial cell adhesion, nuclear division, nucleotide biosynthesis, ribosome biogenesis and assembly, and mitotic spindle assembly. Lastly, Post Spawning to Recrudescence transition was characterized by gene networks associated with egg activation, epithelial to mesenchymal transition, germinal center formation, membrane fluidity, and sperm cell adhesion. All pathways are given in S3 Table.

**Table 3:** Pathways identified as differentially expressed relative to the stage before during RBD testis development.

Developing to Pre-Spawning	Pre-spawning to Spawning	Spawning to Post Spawning	Post Spawning-Recrudescence
action potential duration	amino acid import	actomyosin based movement	blood clotting
beta selection	acid secretion	B-cell response	cytoskeleton organization and biogenesis
cross priming	antibody-dependent cell-mediated cytotoxicity (ADCC)	chromosome condensation	egg activation
gene silencing	agglutination	endothelial cell adhesion	embryo implantation
hypothalamus function	artery dilation	inflammatory response	epithelial to mesenchymal transition
immune cell chemotaxis	B-cell activation	mast cell degranulation	germinal center formation
innate immune response	bile acid metabolism	megakaryocyte differentiation	immune system activation
Leukocyte cell adhesion	biliary flow	mitotic spindle assembly	JNK cascade
lipid transport	branching morphogenesis	neutrophil adhesion	membrane fluidity
lung function	calcium mobilization	neutrophil chemotaxis	microglial activation
lymphocyte chemotaxis	calcium-mediated signaling	nuclear division	mRNA metabolism
lymphocytic blood cell differentiation	endothelial cell production	nucleotide biosynthesis	muscle regeneration
macrophage response	extrinsic pathway of apoptosis	ribosome biogenesis and assembly	response to cocaine
membrane ruffling	glycogenesis	synapsis	secretory pathway
Mitochondrial Ca2+ transport	H+ homeostasis/export	synaptic vesicle exocytosis	sperm cell adhesion
monocyte chemotaxis	habituation	vascularization	
mRNA stabilization	intracellular signaling cascade		
neuron development	isotype switching		
NO biosynthesis	K+ import/homeostasis		
protein nucleus import	killing of inflammatory cells		
response to osmotic stress	LDL oxidation		
response to wounding	lipid export		
sperm cell function	lung blood flow		
stem cell maintenance	mevalonate pathway		
T-cell response	micturition		
T-helper lymphocyte response	monocyte recruitment		
	mucosal permeability		
	natriuresis		
	neural crest cell differentiation		
	ontogeny		
	pigment biosynthesis		
	platelet activation		
	protein-protein cross-linking via L-cystine		
	regulation of cell volume		
	renal reabsorption		
	salinity response		
	saliva secretion		
	Sertoli cell proliferation		
	sperm capacitation		
	sperm motility		
	striatal dopamine release		
	ureteric bud branching		

Each pathway is unique to the indicated transition. The complete list of pathways is presented in S3 Table. Bold and italic pathways indicate immune-related processes.

### Targeted qPCR analysis

Targeted qPCR analysis was conducted on genes of interest that were determined to be important for male reproduction in a previous study [14]. In total, 18 genes listed in Table 4 were analyzed using real-time PCR. None of our genes of interest were found in the “wave” shape of clusters 13, 17 and 18 (Fig 7). These included steroid hormone receptors, enzymes involved in steroid biosynthesis, genes related with spermatogenesis and female sex differentiation, and genes that are indicative of stress and biomarkers of exposure to endocrine disrupting compounds.

**Table 4:** Normalized fold change levels ± standard error for *esr1*, *esrb*, *ar*, *cyp17α*, *cyp19α*, *spag*, *spata4*, *dmrt1*, *sox9*, *foxl2*, *hps90*, *hsp1*, *hsp70*, *nanos3*, *cdca8*, *surviving*, *fabp*, and *zp* analyzed using real-time PCR in male gonads over the different stages of testis development. Differences among treatment groups (p < 0.01) for each gene are denoted with different letters; outliers were included in the analysis.

Gene	Recrudescence (7)	Developing (7)	Pre-spawning (8)	Spawning (6)	Post-spawning (8)
esr1	1.75 ± 0.45 b	2.52 ± 0.80 b	1.000 ± 0.31 b	2.51 ± 0.72 ab	11.56 ± 4.10 a
esrb	0.75 ± 0.14 b	0.87 ± 0.18 b	1.01 ± 0.14 b	1.71 ± 0.27 b	6.06 ± 1.41 a
ar	1.00 ± 0.27 b	1.39 ± 0.20 b	1.68 ± 0.15 b	1.48 ± 0.21 b	6.99 ± 1.59 a
cyp17α	1.00 ± 0.16 b	1.94 ± 0.31 ab	4.03 ± 0.70 a	2.80 ± 0.36 a	2.94 ± 0.48 a
cyp19α	1.54 ± 0.76	2.80 ± 1.70	1.00 ± 0.19	0.87 ± 0.12	3.36 ± 0.82
spag	22.98 ± 5.82 a	8.01 ± 1.14 a	22.78 ± 4.21 a	27.13 ± 7.72 a	1.00 ± 0.34 b
spata4	3.76 ± 0.72 a	1.00 ± 0.17 b	4.14 ± 0.59 a	5.20 ± 084 a	1.10 ± 0.11 b
dmrt1	1.00 ± 0.25 b	1.91 ± 0.39 ab	1.11 ± 0.13 ab	1.16 ± 0.13 ab	3.43 ± 0.68 a
sox9	0.80 ± 0.18 b	0.84 ± 0.16 b	1.00 ± 0.13 b	0.95 ± 0.17 b	19.74 ± 3.19 a
foxl2	1.00 ± 0.29	3.10 ± 1.05	2.95 ± 0.75	1.86 ± 0.55	4.32 ± 1.58
hsp90	1.35 ± 0.30 b	1.00 ± 0.21 b	2.02 ± 0.24 ab	1.61 ± 0.22 ab	4.87 ± 1.28 a
hsbp1	2.34 ± 0.38 ab	1.00 ± 0.26 b	3.54 ± 0.55 a	4.03 ± 0.99 a	1.20 ± 0.33 b
hsp70	1.14 ± 0.20	1.00 ± 0.13	1.97 ± 0.39	1.96 ± 0.23	2.04 ± 0.50
nanos3	1.00 ± 0.16 b	1.26 ± 0.21 b	0.94 ± 0.12 b	1.14 ± 0.24 b	4.39 ± 0.55 a
cdca8	1.13 ± 0.36 b	1.00 ± 0.25 b	0.76 ± 0.16 b	1.11 ± 0.28 b	27.07 ± 5.70 a
surviving	2.16 ± 0.21 bc	4.14 ± 0.63 b	1.00 ± 0.24 b	1.27 ± 0.30 bc	23.52 ± 5.85 a
fabp	2.20 ± 0.74 b	6.54 ± 1.93 a	2.45 ± 0.66 ab	1.00 ± 0.18 b	1.48 ± 0.36 b
zp	1.87 ± 0.47 ab	9.20 ± 3.55 a	1.54 ± 0.57 b	1.00 ± 0.14 b	2.36 ± 1.23 b

The expression of *esr1* did not change in individuals collected from the recrudescence stage to the spawning stage; however, there was a significant peak in *esr1* expression at the post-spawning season (p = 0.004). The same pattern of expression was observed for both *esrb* and *ar*.

Since spermatogenesis is highly controlled by steroid hormones, there was a focus in this study on the expression of genes involved in steroidogenesis. *Cyp17a* was more abundant during the pre-spawning, spawning, and post-spawning seasons, and decreased significantly in individuals collected during recrudescence. Lastly, *cyp19a*, the enzyme responsible for the biosynthesis of 17β-estradiol from testosterone, did not show any significant changes over the different stages of testis development (p = 0.086).

We compared qPCR analysis with the K-means cluster analysis to determine if the qPCR data showed the same changes in abundance as those data collected by the microarray analysis, and this was indeed the case. For example, Cluster 7 (Fig 7) contained the expression pattern of *spata4*, and cluster 12 (Fig 7) contained the expression pattern of *spag* (*sperm associated antigen 1*). S*pag* and *spata4* (*spermatogenesis associated 4*) showed similar expression patterns over testis development, and each transcript was abundant in testes at recrudescence, pre-spawning and spawning season, and was decreased in individuals collected during the developing (winter time) and post-spawning seasons (Table 4). *Spata4* was included in cluster 7 (Fig 7) following the k-mean cluster analysis, and there was a significant decrease in mRNA abundance during developing and post-spawning stages (p < 0.001). *Spag* belonged to a different cluster 12 (Fig 7), but there was also a significant decrease in abundance during the post-spawning season. *Dmrt1* on the other hand, showed high expression during the post-spawning season, with a decrease in expression in the recrudescence season (p = 0.001). Genes related to oogenesis were also investigated in the testes over a breeding season because there is evidence that these genes are increased during intersex [14]. Female transcription factors *sox9* and *foxl2* were examined in the testis. *Sox9* and *foxl2* were below the detection limits of the microarray data, so we could not validate the microarray data with these genes. Interestingly, qPCR data showed that *sox9* significantly increased at the post-spawning stage compared to the other stages, and no significant differences in the expression of *foxl2* over the different stages of development was observed (Table 4).

Genes related to general stress responses also showed different patterns of expression over the year in the testis, while other genes were localized to clusters 10 and 11 (Fig 7). Transcripts such as *hsp90* showed an increase in mRNA abundance in recrudescent males towards the post-spawning stage (p = 0.001), while *hsbp1* was most abundant in the testis during the pre-spawning and spawning seasons (Table 4). A significant decrease in *hsbp1* was detected in males collected in the winter period and the post-spawning season (p < 0.001). No difference in *hsp70* abundance over the different stages was detected (p = 0.076).

Cluster 2 contained genes that showed the lowest transcript abundance at pre-spawning and spawning seasons, with a peak at the post-spawning season (Fig 7). This cluster included *nanos3*, *cdca8*, and *survivin*, each of which were evaluated at the individual level with qPCR (Table 4). The trend in the abundance change was the same for both the microarray and real-time PCR data. Interestingly, *cdca8* and *survivin* interact with each other at the protein level [21] and these two transcripts showed comparable expression patterns over development in the testis.

Fig 7 also depicts some clusters that contain genes with a major peak in expression during the developing season. Transcript levels of *fabp* showed the highest amount in testis from individuals collected during the developing season, and showed a significant decrease in individuals collected at spawning, post-spawning and in recrudescent individuals (cluster 5; p = 0.001). Lastly, isoforms of zona pellucida (*zp*) belonged to cluster 5 and cluster 11 (Fig 7). The qPCR data demonstrated that *zp* increased in the testis of individuals at the developing stage (p = 0.002, Table 4).

## Discussion

Rainbow darter spermatogenesis occurs over an annual cycle [22]. In this study, we examined 5 defined stages of the male reproductive cycle at the molecular level in order to better understand the mechanisms underlying testis development. The somatic indexes evaluated (k, GSI and LSI) showed expected patterns as previously reported by Tetreault in 2013 [23], confirming that the wild fish collected for the molecular analysis had the expected somatic changes over the year of monitoring. Noteworthy was that we observed the same pattern of change between the LSI and the gene cluster 4, suggesting that genes in that cluster are related to LSI. GO terms represented in cluster 4 include an enrichment of genes related to lipid pathways and further investigation is warranted to describe this relationship.

A negative relationship was detected between the percent of spermatozoa and the percent of spermatocytes. This was expected since the spermatogenesis cycle involves the developmental process during which a small number of diploid spermatogonia stem cells produce a large number of highly differentiated spermatozoa carrying a haploid, recombined genome [3]. There are two major developmental steps between spermatocytes and spermatozoa; the meiotic phase with the primary and secondary spermatocytes and the spermiogenic phase with the haploid spermatids emerging from meiosis and differentiating–without further proliferation–into motile, flagellated genome vectors, the spermatozoa. Thus, the percent of spermatozoa is dependent upon the percent of spermatocytes. Furthermore, it was hypothesized that there would be a relationship between the spermatogenic cell proportion and GSI (since both endpoints increase with development); however this was not observed in RBD. We were unable to locate any literature on multi-spawning, small-bodied fish that demonstrated the relationship between spermatogenic cells and GSI. In addition, no relationship was found between the proportion of spermatogonia and the proportion of any other cell type, since the mitotic phase can develop multiple generations of cells. A lack of a relationship between spermatogonia and the proportion of any other cell has also been reported in other fishes with an annual reproductive cycle, such as the dojo loach (*Misgurnus anguillicaudatus*) [24], and greenside darter (*Etheostoma blennioides*) [25].

Cluster analysis using gene expression grouped the individuals by stages. The analysis revealed that males in recrudescence, spawning and pre-spawning shared expression patterns that were more similar to each other when compared to males at the developing and post-spawning stages (Fig 5). This result was confirmed by the PCA analysis (Fig 6). Expression data over a reproductive season are not widely available for other male teleosts fishes. In female largemouth bass [11] it was demonstrated that, based on global gene expression analysis, fish in primary growth stages formed a unique expression clade while individuals in secondary growth stages also formed a distinct clade. The expression profile for atresia, the active reabsorption of oocytes in the ovary that are not ovulated, was most different when compared to all other ovarian stages in the semi-synchronous spawner. These data are consistent with male RBD and, as expected, GSEA revealed that there was an increased prevalence of genes related with phagocytosis at the post-spawning level (Fig 8), when the non-released cells are absorbed, suggesting that this process is highly associated with the stage of development of the cells.

Increased abundance of genes in males collected during recrudescence, pre-spawning, and spawning were located in cluster 7 (as well as clusters 1, 9 and 12). SNEA revealed that genes related to meiosis, spermatogenesis, and spermatid development were preferentially expressed at recrudescence, pre-spawning and spawning stages. Many gene networks related to sperm maturation, for example Sertoli cell proliferation, sperm capacitation, sperm motility, and spermatid development decreased 20–30% in expression when progressing from the developing stage towards post-spawning, suggesting that early on in the testis, there is increased activity to prepare the sperm. Similar pathways were found to be differentially expressed at the same corresponding stage in the fathead minnow (*Pimephales promelas*) testis proteome [26] and the rainbow trout (*Oncorhynchus mykiss*) testis transcriptome [27]. The other stage of interest is the one that is dominated by early stages of spermatogenic cell development (i.e. post-spawning) (clusters 17 and 18; Fig 7). Noteworthy was that there were a number of gene networks related to the immune system that were differentially expressed with advanced stages of reproduction, suggesting that these networks play an important role in male reproduction. For example when the testis transitions from developing to pre-spawning stages, gene networks for T-cell and T-helper lymphocyte responses were decreased ~20%. However, networks for monocyte recruitment and platelet activation were increased in expression, moving from prespawning to spawning stages. This trend continued into post-spawning, and there was a global up-regulation of gene networks related to immune function in the testis, moving from spawning to post spawning stages. These included B-cell response, immune response, and neurtophil adhesion gene networks among many others (S3 Table). In addition, SNEA identified several cell processes that are activated in the transition from spawning to post-spawning stage of development. Those processes are then suppressed as the testis enters recrudescence and begins a new maturation cycle. For example, phagocytosis increased 1.73 fold change (p = 0.001) from spawning to post-spawning; spermatozoa phagocytosis is a process that plays a significant role in testis development, mainly during the regression stage [28].

Interestingly, the development of eggs and sperm, while very different processes, share some common patterns in gene expression during gametogenesis. Cluster 15 contained genes related to gonad development. For example, *cdc20* (a gene present in cluster 15; Table 4) has been shown to have higher expression in gonads than other tissues (kidney, brain and whole body) in zebrafish [29]. Expression data support the hypothesis that this transcript plays an important role in the proliferation of spermatogenic tissue in RBD. In addition, other pathways that peak in females as well as in males in the early stage of gonad development are genes related to the immune system. Martyniuk et al., [11] showed the same peak in the expression of the immune system in early follicular growth of largemouth bass (*Micropterus salmoides*). As stated previously, genes included in cluster 10 and 11 showed the same pattern of expression over the different state of developments. Those clusters are enriched for GO terms related to nuclear cell function and also genes related with cell division and cell cycle processes (i.e. cell proliferation, cell growth, cell cycle, cell death SNEAs), in RBD, thus the different bioinformatics techniques converged in similar themes for pathways (Tables 1 and 2). In addition, similar cell division process findings were reported in ovaries from striped bass (*Morone saxatilis*) [30], and largemouth bass [11]. Thus, there are likely conserved molecular signaling cascades initiated during gamete maturation in male and female fishes.

We measured a number of genes by qPCR for several sex specific genes during testis development. Interestingly, many genes associated with female sex differentiation were significantly increased in the males at post-spawning season (i.e. *esr1*, *sox9*, *cdca8* and *survivin*; Table 4). It was previously shown that steroid hormone receptors *esr1* and *ar* can be more abundant in ovaries than testis in RBD [15], however this is not the case for *esrb*, in which there was no difference in mRNA levels between ovary and testis [15]. Bahamonde et al., [14] found that genes such as *esr1*, *ar*, *sox9*, *cdca8* and others like *foxl2* were significantly increased in intersex individuals. This might suggest a sensitive window for the effects of endocrine disrupting compounds (EDCs) on male fish, and may be related to the subsequent development of intersex.

It has been possible to generate the intersex condition in medaka (*Oryzias latipes*) in the laboratory [31,32], but it appears as though it is not as straightforward for other fish species [33]. In the field, roach (*Rutilus rutilus*) germ cell disruption can be substantial [34]. The authors state that the lack of disruption in the laboratory might be due to the fact that the municipal wastewater effluents (MWWE) was not estrogenic or that the germ cell disruption is a consequence of prolonged, chronic exposure [33]. Conversely, the early stages of testis differentiation may be most sensitive to endocrine disruptors and endogenous hormones, and the testis can be converted to an “ovary” most readily at this point in time. This is based on our data that show there are higher expression levels of androgen and estrogen receptors in this stage in rainbow darter testis compared to more advanced testicular stages. Based on the molecular responses observed here, future studies into the induction of intersex condition should investigate the post-spawning season and perhaps, the immune system, as a novel mechanism of intersex.

## Summary

The post-spawning period was the dominant phase that separated individuals based upon gene expression patterns (i.e. was the most different). The post-spawning stage contained gene networks related to apoptosis, cell death, and cell cycle, as well as genes for RNA binding and ribosomal units (clusters 2, 10, and 11), spermatoproteosome complex (cluster 16), and genes involved in stress responses, for example *hsp90* and *hsp1* (cluster 10). Subnetwork enrichment analysis revealed that during the post-spawning season, genes related to oxidative stress and immune responses (cluster 13, 17 and 18) as well as ATPase binding and apoptosis were differentially expressed compared to other stages. There were also a wide variety of genes that showed lower relative expression at post-spawning compared to the other stages. These included genes related to meiosis, cytokinesis and spermatogenesis (cluster 7 and 15), synaptic transmission (cluster 7), protein localization to organelles (cluster 15), and cellular processes involved in reproduction (cluster 12). Developing males showed increased mRNA abundance of genes associated with lipid mobilization (cluster 4), and *fabp* which is involved during the lipid mobilization process. *Fabp* was most expressed at this period based upon the real-time PCR data. This study increases our understanding of testis development in teleost fish and identifies new genes and processes that may be useful in understanding exposure to hormones and EDCs.

## Supporting Information

S1 Table Water quality data provided by The Grand River Conservation Authority. Water Temperature (deg C), pH, Specific Conductance (μS/cm), Dissolved oxygen (mg/L), and turbidity (NTU) was measured hourly (when it was possible) from may 11^th^, 2011 to June 1^st^, 2012.
(XLSX)

S2 Table List of genes included in each one of the 18 different patterns of gene expression determined by the k-mean analysis. (XLSX)

S3 Table The complete list of pathways identified as differentially expressed relative to the stage before during RBD testis development. (XLSX)

## Abbreviations

ACTR2: ARP2 actin-related protein 2 homolog (yeast)
AGT: angiotensinogen (serpin peptidase inhibitor, clade A, member 8)
AIF1: allograft inflammatory factor 1
ANXA5: annexin A5
APOH: apolipoprotein H (beta-2-glycoprotein I)
B2M: beta-2-microglobulin
B4GALT1: UDP-Gal:betaGlcNAc beta 1,4- galactosyltransferase, polypeptide 1
BET1: blocked early in transport 1 homolog (S. cerevisiae)
BET1L: blocked early in transport 1 homolog (S. cerevisiae)-like
BLOC1S1: biogenesis of lysosomal organelles complex-1, subunit 1
BTK: Bruton agammaglobulinemia tyrosine kinase
C1QBP: complement component 1, q subcomponent binding protein
CASP3: caspase 3, apoptosis-related cysteine peptidase
CD59 molecule: complement regulatory protein
CDC42: cell division cycle 42 (GTP binding protein, 25kDa)
CDH2: cadherin 2, type 1, N-cadherin (neuronal)
CDK1: cyclin-dependent kinase 1
CDK5: cyclin-dependent kinase 5
CDK6: cyclin-dependent kinase 6
CDKN2D: cyclin-dependent kinase inhibitor 2D (p19, inhibits CDK4)
CETN1: centrin, EF-hand protein, 1
CFH: complement factor H
CHN1: chimerin (chimaerin) 1
CIRH1A: cirrhosis, autosomal recessive 1A (cirhin)
CLDN7: claudin 7
CMA1: chymase 1, mast cell
CNR1: cannabinoid receptor 1 (brain)
CNTF: ciliary neurotrophic factor
CTPS: CTP synthase
CTSS: cathepsin S
DAZL: deleted in azoospermia-like
DHFR: dihydrofolate reductase
DYDC1: DPY30 domain containing 1
EBNA1BP2: EBNA1 binding protein 2
EDA: ectodysplasin A
EIF2AK1: eukaryotic translation initiation factor 2-alpha kinase 1
EIF4EBP1: eukaryotic translation initiation factor 4E binding protein 1
EMP2: epithelial membrane protein 2
EPO: erythropoietin
F11R: F11 receptor
F2RL1: coagulation factor II (thrombin) receptor-like 1
FAS: Fas (TNF receptor superfamily, member 6)
FAU: Finkel-Biskis-Reilly murine sarcoma virus (FBR-MuSV) ubiquitously expressed
FTH1: ferritin, heavy polypeptide 1
FZR1: fizzy/cell division cycle 20 related 1 (Drosophila)
G6PD: glucose-6-phosphate dehydrogenase
GAPDH: glyceraldehyde-3-phosphate dehydrogenase
GH1: growth hormone 1
GLRX: glutaredoxin (thioltransferase)
GMFG: glia maturation factor, gamma
GMNN: geminin, DNA replication inhibitor
GNAQ: guanine nucleotide binding protein (G protein), q polypeptide
GNAS: GNAS complex locus
HMGB1: high mobility group box 1
HPRT1: hypoxanthine phosphoribosyltransferase 1
HRSP12: heat-responsive protein 12
HSP90B1: heat shock protein 90kDa beta (Grp94), member 1
HSPA8: heat shock 70kDa protein 8
HSPE1: heat shock 10kDa protein 1 (chaperonin 10)
IDH2: isocitrate dehydrogenase 2 (NADP+), mitochondrial
IGF2: insulin-like growth factor 2 (somatomedin A)
IL10: interleukin 10
IL5: interleukin 5 (colony-stimulating factor, eosinophil)
INS: insulin
KIT: v-kit Hardy-Zuckerman 4 feline sarcoma viral oncogene homolog
LEP: leptin
LGALS3: lectin, galactoside-binding, soluble, 3
MAL: mal, T-cell differentiation protein
MAPK1: mitogen-activated protein kinase 1
MAPK14: mitogen-activated protein kinase 14
MAPKAPK2: mitogen-activated protein kinase-activated protein kinase 2
MBL2: mannose-binding lectin (protein C) 2, soluble
MBP: myelin basic protein
MDK: midkine (neurite growth-promoting factor 2)
MEIG1: meiosis expressed gene 1 homolog (mouse)
MIF: macrophage migration inhibitory factor (glycosylation-inhibiting factor)
MMP2: matrix metallopeptidase 2 (gelatinase A, 72kDa gelatinase, 72kDa type IV collagenase)
MOG: myelin oligodendrocyte glycoprotein
MSRB2: methionine sulfoxide reductase B2
MYD88: myeloid differentiation primary response gene (88)
NEU1: sialidase 1 (lysosomal sialidase)
NFE2L2: nuclear factor (erythroid-derived 2)-like 2
NIP7: nuclear import 7 homolog (S. cerevisiae)
NKX2-1: NK2 homeobox 1
NOP58: NOP58 ribonucleoprotein homolog (yeast)
NPM1: nucleophosmin (nucleolar phosphoprotein B23, numatrin)
NPY: neuropeptide Y
NR3C1: nuclear receptor subfamily 3, group C, member 1 (glucocorticoid receptor)
OSGEP: O-sialoglycoprotein endopeptidase
PACRG: PARK2 co-regulated
PDIA3: protein disulfide isomerase family A, member 3
PIN4: protein (peptidylprolyl cis/trans isomerase) NIMA-interacting, 4 (parvulin)
POMP: proteasome maturation protein
PRDX6: peroxiredoxin 6
PSMB8: proteasome (prosome, macropain) subunit, beta type, 8 (large multifunctional peptidase 7)
PSMB9: proteasome (prosome, macropain) subunit, beta type, 9 (large multifunctional peptidase 2)
PSME1: proteasome (prosome, macropain) activator subunit 1 (PA28 alpha)
PYCR1: pyrroline-5-carboxylate reductase 1
RAB13: member RAS oncogene family
RAB27A: RAB27A, member RAS oncogene family
RAB7A: member RAS oncogene family
RAB8A: member RAS oncogene family
RAN: member RAS oncogene family
RAP1A: member of RAS oncogene family
RAP1B: member of RAS oncogene family
RB1: retinoblastoma 1
RGS18: regulator of G-protein signaling 18
RHOA: ras homolog gene family, member A
RPF2: ribosome production factor 2 homolog (S. cerevisiae)
RPL10: ribosomal protein L10
RPL11: ribosomal protein L11
RPL14: ribosomal protein L14
RPL23: ribosomal protein L23
RPL23A: ribosomal protein L23a
RPL27: ribosomal protein L27
RPL28: ribosomal protein L28
RPL3: ribosomal protein L3
RPS10: ribosomal protein S10
RPS12: ribosomal protein S12
RPS19: ribosomal protein S19
RPS2: ribosomal protein S2
RPS3: ribosomal protein S3
RPS6: ribosomal protein S6
RPS9: ribosomal protein S9
RRS1: RRS1 ribosome biogenesis regulator homolog (S. cerevisiae)
RSL24D1: ribosomal L24 domain containing 1
RSPH1: radial spoke head 1 homolog (Chlamydomonas)
S100A8: S100 calcium binding protein A8
S100A9: S100 calcium binding protein A9
SAA1: serum amyloid A1
SBDS: Shwachman-Bodian-Diamond syndrome
SEC22B: SEC22 vesicle trafficking protein homolog B (S. cerevisiae) (gene/pseudogene)
SERPINA1: serpin peptidase inhibitor, clade A (alpha-1 antiproteinase, antitrypsin), member 1
SERPINE1: serpin peptidase inhibitor, clade E (nexin, plasminogen activator inhibitor type 1), member 1
SKAP2: src kinase associated phosphoprotein 2
SLPI: secretory leukocyte peptidase inhibitor
SPARC: secreted protein, acidic, cysteine-rich (osteonectin)
SPI1: spleen focus forming virus (SFFV) proviral integration oncogene spi1
STIM1: stromal interaction molecule 1
STX4: syntaxin 4
STXBP2: syntaxin binding protein 2
SUPT4H1: suppressor of Ty 4 homolog 1 (S. cerevisiae)
SURF6: surfeit 6
TBPL1: TBP-like 1
TCP1: t-complex 1
TF: transferrin
TMSB4X: thymosin beta 4, X-linked
TNFRSF1B: tumor necrosis factor receptor superfamily, member 1B
TSPO: translocator protein (18kDa)
TSSK1B: testis-specific serine kinase 1B
UCK2: uridine-cytidine kinase 2
UCN: urocortin
VAMP1: vesicle-associated membrane protein 1 (synaptobrevin 1)
VAMP3: vesicle-associated membrane protein 3 (cellubrevin)
VAMP8: vesicle-associated membrane protein 8 (endobrevin)
VAPA: VAMP (vesicle-associated membrane protein)-associated protein A, 33kDa
VTI1A: vesicle transport through interaction with t-SNAREs homolog 1A (yeast)
VTI1B: vesicle transport through interaction with t-SNAREs homolog 1B (yeast)
WDR1: WD repeat domain 1
YBX1: Y box binding protein 1.

## References

[1] Barrett TJ, Munkittrick KR. Seasonal reproductive patterns and recommended sampling times for sentinel fish species used in environmental effects monitoring programs in Canada. Environ Rev. 2010;18: 115–135. 10.1139/A10-004

[2] Tetreault GR, Brown CJ, Bennett CJ, Oakes KD, McMaster ME, Servos MR. Fish community responses to multiple municipal wastewater inputs in a watershed: Fish community changes near wastewater effluent outfalls. Integr Environ Assess Manag. 2013;9: 456–468. 10.1002/ieam.1364 22976948

[3] Schulz RW, de França LR, Lareyre J-J, LeGac F, Chiarini-Garcia H, Nobrega RH, et al Spermatogenesis in fish. Gen Comp Endocrinol. 2010;165: 390–411. 10.1016/j.ygcen.2009.02.013 19348807

[4] Miura T, Miura CI. Molecular control mechanisms of fish spermatogenesis. Fish Physiol Biochem. 2003;28: 181–186.

[5] Vlaming VL. Environmental control of teleost reproductive cycles: a brief review. J Fish Biol. 1972;4: 131–140.

[6] Munro AD, Scott AP, Lam TJ. Reproductive seasonality in teleosts: Environmental influences. Florida: CRC Press; 1990.

[7] Kime DE. Endocrine Disruption in Fish. Springer 1998.

[8] Tetreault GR. The Response of Wild Fish to Municipal Wastewater Effluent Exposures at Sites in Canada [Internet]. University of Waterloo. 2012. Available: http://libdspace.uwaterloo.ca/handle/10012/6621

[9] Bobe J, Montfort J, Nguyen T, Fostier A. Identification of new participants in the rainbow trout (*Oncorhynchus mykiss*) oocyte maturation and ovulation processes using cDNA microarrays. Reprod Biol Endocrinol. 2006;4: 39 10.1186/1477-7827-4-39 16872517 PMC1570352

[10] Villeneuve DL, Garcia-Reyero N, Martinović D, Cavallin JE, Mueller ND, Wehmas LC, et al Influence of ovarian stage on transcript profiles in fathead minnow (*Pimephales promelas*) ovary tissue. Aquat Toxicol. 2010;98: 354–366. 10.1016/j.aquatox.2010.03.006 20363515

[11] Martyniuk CJ, Prucha MS, Doperalski NJ, Antczak P, Kroll KJ, Falciani F, et al Gene expression networks underlying ovarian development in wild largemouth bass (*Micropterus salmoides*). Foulkes NS, editor. PLoS ONE. 2013;8: e59093 10.1371/journal.pone.0059093 23527095 PMC3604104

[12] Hook SE, Nagler JJ, Cavileer T, Verducci J, Liu Y, Hayton W, et al Relationships between the transcriptome and physiological indicators of reproduction in female rainbow trout over an annual cycle. Environ Toxicol Chem. 2011;30: 309–318. 10.1002/etc.407 21086553

[13] Sambroni E, Lareyre J-J, Le Gac F. Fsh controls gene expression in fish both independently of and through steroid mediation. Schlatt S, editor. PLoS ONE. 2013;8: e76684 10.1371/journal.pone.0076684 24194844 PMC3806798

[14] Bahamonde PA, McMaster ME, Servos MR, Martyniuk CJ, Munkittrick KR. Molecular pathways associated with the intersex condition in rainbow darter (*Etheostoma caeruleum*) following exposures to municipal wastewater in the Grand River basin, ON, Canada. Part B. Aquat Toxicol. 2015;159: 302–316. 10.1016/j.aquatox.2014.11.022 25542366

[15] Bahamonde PA, Tetreault GR, McMaster ME, Servos MR, Martyniuk CJ, Munkittrick KR. Molecular signatures in rainbow darter (*Etheostoma caeruleum*) inhabiting an urbanized river reach receiving wastewater effluents. Aquat Toxicol. 2014;148: 211–220. 10.1016/j.aquatox.2014.01.010 24513783

[16] Rozen S, Skaletsky H. Primer3 on the WWW for General Users and for Biologist Programmers. Methods Mol Biol. 2000;132: 365–386. 10547847 10.1385/1-59259-192-2:365

[17] Bustin SA, Benes V, Garson JA, Hellemans J, Huggett J, Kubista M, et al The MIQE guidelines: Minimum information for publication of quantitative Real-Time PCR experiments. Clin Chem. 2009;55: 611–622. 10.1373/clinchem.2008.112797 19246619

[18] Martyniuk CJ, Feswick A, Spade DJ, Kroll KJ, Barber DS, Denslow ND. Effects of acute dieldrin exposure on neurotransmitters and global gene transcription in largemouth bass (Micropterus salmoides) hypothalamus. NeuroToxicology. 2010;31: 356–366. 10.1016/j.neuro.2010.04.008 20438755 PMC2882520

[19] Bolstad BM, Irizarry RA, AAstrand M, Speed TP. A comparison of normalization methods for high density oligonucleotide array data based on variance and bias. Bioinformatics. 2003;19: 185–193. 12538238 10.1093/bioinformatics/19.2.185

[20] Subramanian A, Tamayo P, Mootha VK, Mukherjee S, Ebert BL, Gillette MA, et al Gene set enrichment analysis: a knowledge-based approach for interpreting genome-wide expression profiles. Proc Natl Acad Sci U S A. 2005;102: 15545–15550. 10.1073/pnas.0506580102 16199517 PMC1239896

[21] Nogueira-Ferreira R, Vitorino R, Ferreira-Pinto MJ, Ferreira R, Henriques-Coelho T. Exploring the role of post-translational modifications on protein–protein interactions with survivin. Arch Biochem Biophys. 2013;538: 64–70. 10.1016/j.abb.2013.07.027 23938875

[22] Fuller RC. Fecundity estimates for rainbow darters, *Etheostoma caeruleum*, in southwestern Michigan. Ohio J Sci. 1998;98: 2–5.

[23] Tetreault GR, Bennett CJ, Servos MR, McMaster ME. Optimization of effects-assessment of greenside darter (*Etheostoma blennioides*) exposed to tertiary treated municipal wastewater based on seasonal changes of reproductive endpoints: Optimization of effects-assessment of darters. Environ Toxicol Chem. 2014;33: 1077–1089. 10.1002/etc.2526 24459008

[24] Kiros S, Aoki J, Park C-B, Soyano K. Annual changes in testicular development and plasma sex steroids in the captive male dojo loach *Misgurnus anguillicaudatus*. Ichthyol Res. 2011;58: 217–224. 10.1007/s10228-011-0213-7

[25] Tetreault GR, Bennett CJ, Cheng C, Servos MR, McMaster ME. Reproductive and histopathological effects in wild fish inhabiting an effluent-dominated stream, Wascana Creek, SK, Canada. Aquat Toxicol. 2012;110–111: 149–161. 10.1016/j.aquatox.2012.01.004 22307007

[26] Martyniuk CJ, Alvarez S. Proteome analysis of the fathead minnow (*Pimephales promelas*) reproductive testes. J Proteomics. 2013;79: 28–42. 10.1016/j.jprot.2012.11.023 23234800

[27] Mazurais D, Montfort J, Delalande C, Gac FL. Transcriptional analysis of testis maturation using trout cDNA macroarrays. Gen Comp Endocrinol. 2005;142: 143–154. 10.1016/j.ygcen.2005.02.018 15862558

[28] Cruz-Landim C, Abdalla FC, Cruz-Höfling MA. Morphological changes of Sertoli cells during the male reproductive cycle of the teleost *Piaractus mesopotamicus* (Holmberg, 1887). Braz J Biol. 2005;65: 241–249. 16097726 10.1590/s1519-69842005000200007

[29] Sreenivasan R, Cai M, Bartfai R, Wang X, Christoffels A, Orban L. Transcriptomic analyses reveal novel genes with sexually dimorphic expression in the zebrafish gonad and brain. Butler G, editor. PLoS ONE. 2008;3: e1791 10.1371/journal.pone.0001791 18335061 PMC2262149

[30] Reading BJ, Chapman RW, Schaff JE, Scholl EH, Opperman CH, Sullivan CV. An ovary transcriptome for all maturational stages of the striped bass (*Morone saxatilis*), a highly advanced perciform fish. BMC Res Notes. 2012;5: 111 10.1186/1756-0500-5-111 22353237 PMC3305648

[31] Kang IJ, Yokota H, Oshima Y, Tsuruda Y, Yamaguchi T, Maeda M, et al Effect of 17β-estradiol on the reproduction of Japanese medaka (*Oryzias latipes*). Chemosphere. 2002;47: 71–80. 11996138 10.1016/s0045-6535(01)00205-3

[32] Koger CS, Teh SJ, Hinton DE. Determining the sensitive developmental stages of intersex induction in medaka (*Oryzias latipes*) exposed to 17β-estradiol or testosterone. Mar Environ Res. 2000;50: 201–206. 10.1016/S0141-1136(00)00068-4 11460690

[33] Liney KE, Jobling S, Shears JA, Simpson P, Tyler CR. Assessing the sensitivity of different life stages for sexual disruption in roach (*Rutilus rutilus*) exposed to effluents from wastewater treatment works. Environ Health Perspect. 2005;113: 1299–1307. 10.1289/ehp.7921 16203238 PMC1281270

[34] Jobling S, Nolan M, Tyler CR, Brighty G, Sumpter JP. Widespread sexual disruption in wild fish. Environ Sci Technol. 1998;32: 2498–2506.

